# Seropositivity to Cytomegalovirus, Inflammation, All-Cause and Cardiovascular Disease-Related Mortality in the United States

**DOI:** 10.1371/journal.pone.0016103

**Published:** 2011-02-17

**Authors:** Amanda M. Simanek, Jennifer Beam Dowd, Graham Pawelec, David Melzer, Ambarish Dutta, Allison E. Aiello

**Affiliations:** 1 Department of Epidemiology, Center for Social Epidemiology and Population Health, School of Public Health, University of Michigan, Ann Arbor, Michigan, United States of America; 2 Epidemiology and Biostatistics, School of Public Health, Hunter College, City University of New York (CUNY), CUNY Institute for Demographic Research, New York, New York, United States of America; 3 Second Department of Internal Medicine, Center for Medical Research, ZMF, University of Tuebingen Medical School, Tuebingen, Germany; 4 Epidemiology and Public Health Group, Peninsula Medical School, Exeter, United Kingdom; Lerner Research Institute, Cleveland Clinic, United States of America

## Abstract

**Background:**

Studies have suggested that CMV infection may influence cardiovascular disease (CVD) risk and mortality. However, there have been no large-scale examinations of these relationships among demographically diverse populations. The inflammatory marker C-reactive protein (CRP) is also linked with CVD outcomes and mortality and may play an important role in the pathway between CMV and mortality. We utilized a U.S. nationally representative study to examine whether CMV infection is associated with all-cause and CVD-related mortality. We also assessed whether CRP level mediated or modified these relationships.

**Methodology/Principal Findings:**

Data come from subjects ≥25 years of age who were tested for CMV and CRP level and were eligible for mortality follow-up on December 31^st^, 2006 (N = 14153) in the National Health and Nutrition Examination Survey (NHANES) III (1988–1994). Cox proportional hazard models were used to estimate hazard ratios (HR) and 95% confidence intervals (CI) for all-cause and CVD-related mortality by CMV serostatus. After adjusting for multiple confounders, CMV seropositivity remained statistically significantly associated with all-cause mortality (HR 1.19, 95% CI: 1.01, 1.41). The association between CMV and CVD-related mortality did not achieve statistical significance after confounder adjustment. CRP did not mediate these associations. However, CMV seropositive individuals with high CRP levels showed a 30.1% higher risk for all-cause mortality and 29.5% higher risk for CVD-related mortality compared to CMV seropositive individuals with low CRP levels.

**Conclusions/Significance:**

CMV was associated with a significant increased risk for all-cause mortality and CMV seropositive subjects who also had high CRP levels were at substantially higher risk for both for all-cause and CVD-related mortality than subjects with low CRP levels. Future work should target the mechanisms by which CMV infection and low-level inflammation interact to yield significant impact on mortality.

## Introduction

Cytomegalovirus (CMV) is a highly transmissible and prevalent beta herpesvirus [1], [2]. This pathogen is never cleared from the body, persisting in a number of tissues via hypothesized mechanisms including chronic productive infection and/or latent infection with periodic subclinical reactivation [2], [3]. Recently, CMV has been linked to a variety of chronic diseases with an inflammatory component including cardiovascular disease (CVD) [4]–[7], cancer [8], [9], cognitive decline including vascular dementia [10], [11] and functional impairment [12]–[14].

Several mechanisms have been hypothesized to link CMV infection and CVD in both human and animal studies [15]–[20]. CMV antigen and DNA have been identified in atherosclerotic vessels of the human cardiovascular system [21]–[23] and murine models suggest an inflammatory link with CVD progression [19], [20]. It has been hypothesized that CMV either directly infects the vessels of the heart and replicates at low levels, or is delivered to the vessel wall by infected circulating monocytes arriving at sites of cardiovascular injury or inflammation [24]. The presence of CMV in the vessel walls may induce smooth muscle cell proliferation and migration, increased uptake of oxidized low-density lipoprotein and expression of cytokines and chemokines as well as increased procoagulant activity by endothelial cells [16], [17], [25], [26]. CMV may also cause vascular damage without direct invasion as a result of molecular mimicry, whereby viral antigens trigger an immune response cross-reacting on self-peptides expressed on uninfected host tissues [16], [27], [28]. For example, two CMV-derived proteins, UL122 and US28, are homologous to an amino acid sequence at position 153–160 of heat shock protein (HSP) 60 [29], [30]. Thus, infection with CMV may contribute to progression of atherosclerosis and other CVD health outcomes via several mechanisms.

CMV has also been associated with other chronic diseases of aging, including physical impairment, cognitive decline and cancer [8]–[14]. The specific mechanisms responsible for these associations have not been fully elucidated, but are likely to have an immune and inflammatory component. Indeed, CMV seropositivity belongs to a cluster of immune factors constituting an “immune risk profile” associated with all-cause mortality at 2, 4 and 6-year follow-up in elderly Swedes in the OCTO/NONA longitudinal studies [31]–[34]. CMV is a driver of age-associated immune changes in elderly populations which lead to a reduction in the number of naïve T cells available for fighting new infections [34]–[37]. Reactivations or superinfections may result in higher titers of CMV immunoglobulin G (IgG) antibodies and increased levels of pro-inflammatory cytokines such as interleukin-6 (IL-6) and tumor necrosis factor-alpha (TNF-α) [38]–[40]. C-reactive protein (CRP) levels also increase as a consequence of reactivation or leakage of the virus from host cells, via the action of IL-6 produced in the liver [17], [41]–[43]. These inflammatory markers have been linked to both all-cause and CVD-related mortality [44]–[46]. Thus, CMV may impact both CVD-related and all-cause mortality through its affects on chronic inflammatory and immune-related changes seen with aging.

Although not all studies have supported a relationship between CMV and chronic disease outcomes [47], [48], a majority of published studies have reported a significant relationship between CMV and mortality, in conjunction with markers of inflammation [14], [49]–[52]. A recent study by Roberts et al., examined the relationship between CMV antibody titer and all-cause/CVD mortality among elderly Latinos finding that subjects with CMV IgG antibody titers in the highest quartile had 1.43 (95% CI: 1.14, 1.79) times higher risk for all-cause mortality and 1.35 (95% CI: 1.01, 1.80) times higher risk for CVD mortality in models adjusted for age, gender, education and comorbidity index [49]. The authors found the effect of CMV on mortality was partially mediated by a composite measure of two inflammatory markers, TNF-α and IL-6, but not CRP since it was unrelated to mortality in their study population [49]. In contrast, Blankenberg et al. found CMV to be associated with cardiac mortality among persons with existing coronary artery disease (CAD) only in subjects with elevated IL-6 levels (hazard ratio (HR) 3.2, 95% confidence interval (CI): 1.4, 7.3) and not in those without IL-6 elevation, suggesting effect modification by IL-6 in the association between CMV and CVD-related mortality [52]. Also examining the effect of CMV antibody titers on mortality, Strandberg et al. assessed 7-year risk for mortality in a small cohort of community-dwelling 75–90 year-olds with underlying CVD in Finland [50]. The authors found that mortality was significantly greater among subjects in the highest CMV IgG quartile, compared with the lowest quartile and that this association remained significant after controlling for several covariates including CRP, suggesting that CRP did not mediate this pathway [50]. Furthermore, Muhlestein et al. examined the association between seropositivity to CMV, *H. pylori* and *Chlamydia pneumonia* infections, as well as high CRP level and mortality among patients with a mean age of 65 years that were predominantly male and had angiographically demonstrated CAD [51]. Of the three pathogens assessed, only seropositivity to CMV was significantly predictive of mortality. The authors also found that risk for mortality was greatest among CMV seropositive subjects with CRP levels in the highest tertile, suggesting a joint effect of CMV seropositivity and high CRP level on mortality among their sample of individuals with underlying CAD [51].

Even though these studies suggest an association between CMV, inflammation and mortality, they were conducted in predominantly older age populations, of specific gender or race/ethnic groups and/or among participants who have underlying CAD or CVD health conditions. While one cross-sectional study conducted among a U.S. population-based cohort of individuals aged 45 and older found that CMV was associated with reported history of CVD, it remains unclear whether a temporal relationship exists between CMV and mortality in the U.S. population [6]. Moreover, the role of inflammatory markers such as IL-6, TNF-α and CRP as mediators or effect modifiers of the relationship between CMV and mortality is unclear [14], [49]–[52]. Some studies support mediation by these markers, while others suggest effect modification [14], [49]–[52]. Therefore, research examining whether inflammatory markers mediate or modify the relationship between CMV and mortality in the U.S. is needed.

The purpose of this study was to examine whether seropositivity for CMV predicts all-cause as well as CVD-related mortality and whether CRP mediated or modified these relationships in a nationally representative U.S. population of individuals aged 25 and older.

## Methods

### Ethics statement

The University of Michigan Institutional Review Board approved this study HUM00013336.

### Study population

Data come from the National Health and Nutrition Examination Survey (NHANES) III (1988–1994), a population-based, multistage stratified probability survey which collects information on the health and nutrition of the United States civilian noninstitutionalized population. The survey was carried out by the National Center for Health Statistics (NCHS), Centers for Disease Control and Prevention and is meant to be representative of the U.S. population. In addition, we used data from the NHANES III-Linked Mortality file in which the mortality status of NHANES III participants, ≥17 years of age, was determined by probabilistic matching between NHANES III participants and the U.S. National Death Index (NDI) [53].

A total of 33994 subjects were interviewed in NHANES III. Our study sample is limited to subjects that were 25 years of age and older (range 25–90 years of age) at time of examination (N = 15242 (48.7%)), were tested for CMV serostatus and CRP level (N = 14164 (92.9%)) and eligible for mortality follow-up on December 31^st^, 2006 (N = 14153 (99.9%). Forty-eight subjects (0.3%) were excluded from the analyses of CVD-related mortality because their cause of death could not be ascertained.

### Laboratory Analyses

CMV-specific IgG was measured by a commercially available Enzyme Linked Immunosorbent Assay (ELISA) (Quest International, Inc., Miami, FL). Sera with values near the ELISA cutoff were confirmed with a second ELISA assay (bioMerieux, Inc., Durham, NC). If the results from the first two tests disagreed, an Immunofluorescence Assay (IFA) (Bion International, Inc., Park Ridge, IL), was used and results from this test were provided as the final seropositivity test result. The sensitivity and specificity of these tests have been estimated to be 98% and 99%, respectively [1], [54]. Although CMV IgG and IgM antibody titer levels are available in NHANES III, values for subjects over age 49 were top-coded, making them unusable as a predictor of mortality.

Sera collected for the purpose of CRP testing were stored at -70 C and analyzed within 2 months using a modification of the Behring Latex-Enhanced CRP assay on the Behring Nephelometer Analyzer system™ (Behring Diagnostics, Westwood, MA). Both within and between-assay quality control procedures were used and the coefficient of variation was 3.2%–16.1% through the period of data collection. The limit of detection for CRP was 0.3 mg/dL [55].

Sera collected for the purpose of cholesterol testing were frozen and stored at -20 C, then shipped on dry ice within four weeks to the Johns Hopkins University Lipid Research Clinic Laboratory which participates in the Lipid Standardization Program of the Centers for Disease Control and Prevention. Total cholesterol was measured enzymatically on a Hitachi 717 analyzer using a commercially available reagent mixture (Boehringer Manheim Diagnostics, Indianapolis, IN) [55].

### Measures

Results from the CMV IgG antibody tests were dichotomized as seronegative or seropositive based on the ELISA results. Results from the CRP tests were dichotomized as low: <0.3 mg/dL and high: ≥0.3 mg/dL according to commonly used cut-off values thought to have clinical significance for prediction of heart attack or stroke [56]. Combined CMV serostatus and CRP level was categorized as CMV seronegative/low CRP, CMV seronegative/high CRP, CMV seropositive/low CRP and CMV seropositive/high CRP.

Mortality status was obtained primarily from the NDI; however, other sources of mortality status included indication of deceased status from the Social Security Administration, the Centers for Medicare and Medicaid Services, or death certificate review [53]. Cause of death was coded using the International Classification of Diseases, Ninth Revision (ICD-9) up until 1998 and ICD-10 for 1999–2006. All deaths before 1999 were recoded by the NCHS into comparable ICD-10 codes [57]. ICD-10 codes I00-I99 were classified as CVD-related deaths and included, causes of death such as hypertensive heart disease, atherosclerosis including coronary and cerebrovascular disease, heart failure, and aortic aneurysms. Persons who survived the entire follow-up period were administratively censored on December, 31^st^ 2006. Follow-up time for each person was calculated as the difference between the NHANES III examination date and the last known date alive or censored [57]. Persons who died of non-CVD causes were considered censored at the date of death for CVD mortality analysis.

Covariates hypothesized to be potential confounders of the relationship between CMV serostatus and mortality included age, gender, race/ethnicity, country of origin, body mass index (BMI) (kg/m^2^), smoking status, diabetes status and education level, because these factors have been shown to predict both risk for infection and mortality [1], [58]–[62]. Age in years at examination was self-reported and treated as a continuous variable. Gender was dichotomized as female and male. Race/ethnicity was self-reported as non-Hispanic white, non-Hispanic black, Mexican-American or Other. Country of origin was reported as the state or foreign country in which subjects were born and was categorized as U.S. or Other. Education level was chosen as a marker of life course socioeconomic position most likely to precede CMV infection and self-reported as years of education and treated as continuous [63], [64]. BMI (kg/m^2^) was computed from weight and standing height and categorized as BMI <25, 25≤ BMI <30 and BMI ≥30. Smoking status was self-reported and categorized into never (did not smoke 100+ cigarettes in one's lifetime), past (smoked 100+ cigarettes in one's lifetime but do not currently smoke) and current smoker (smoked 100+ cigarettes in one's lifetime and currently smoke). Diabetes was self-reported as whether a doctor ever informed subjects they had diabetes or not and dichotomized as reported or not reported. In addition, use of non-steroidal anti-inflammatory drugs (NSAID) was hypothesized as a confounder in the association between the combined effect of CMV serostatus/CRP level and mortality [65], [66]. Subjects were considered to use NSAIDs if they reported use of prescription drugs in the past month with a primary classification of NSAID according to the Product Information Branch, Center for Drug Evaluation and Research at the U.S. DHHS Food and Drug Administration or if subjects self-reported taking any Advil, Nuprin, Medipren or Ibuprofen in the past month [67].

Total serum cholesterol and hypertension were hypothesized as mediators in the pathway between the combined effect of CMV serostatus/CRP level and CVD-related mortality. Serum total cholesterol level was dichotomized as low (<240 mg/dL) and high (≥240 mg/dL) according to the National Cholesterol Education Program Expert Panel [68] and hypertension was reported as ever having been told by a doctor or health professional that you had hypertension, also called high blood pressure.

### Statistical analyses

Statistical analyses were performed using SAS, version 9.2, with SAS-callable SUDAAN, version 10.0.1 (SAS Institute, Inc., Cary, NC) [69]. All analyses used appropriate weights and adjustments for strata and clustering used in the complex study design used in NHANES III. Bivariate relationships between CMV serostatus, CRP level, all-cause and CVD-related mortality and potential confounders including age, gender, race/ethnicity, country of origin, education level, BMI (kg/m^2^), smoking status, diabetes status and NSAID use were assessed. Covariates were considered confounders based on a priori hypotheses and if they were associated with the exposure and associated with the outcome among the unexposed [70]. T-tests (two-tailed) for difference in means and Pearson chi-square tests of independence for proportions and test for trend among demographic groupings were calculated.

Kaplan-Meier survival curves were plotted for all-cause and CVD-related mortality by CMV serostatus to examine the unadjusted association between CMV serostatus and all-cause/CV-related mortality. Survival time was measured in months since mobile or home examination. Cox proportional hazard models were used to estimate the confounder adjusted HR and 95% CI for the association between CMV serostatus and all-cause/CVD-related mortality first in models adjusted for sociodemographic factors (age, gender, race/ethnicity, country of origin and education level), then, in models additionally adjusted for clinical factors (BMI (kg/m^2^), smoking status and diabetes status and last in models also controlling for CRP level. In order to assess whether CRP level mediated the relationship between CMV and mortality we compared the HR for all-cause and CVD-related mortality, before and after controlling for CRP in the fully adjusted model. Adjusted Wald F statistics were estimated to compare follow-up time in months from exam to death between those CMV seronegative versus CMV seropositive in fully adjusted models.

To assess whether CRP modified the relationship between CMV and all-cause/CVD-related mortality we first examined the crude association between combined CMV serostatus and CRP level by plotting unadjusted Kaplan-Meier survival curves for all-cause and CVD-related mortality by combined CMV serostatus and CRP level over the follow-up period. Survival time was measured in months since mobile or home examination. Next we used Cox proportional hazard models to estimate the confounder adjusted HR and 95% CI for the association between CMV serostatus/CRP level and all-cause/CVD-related mortality first in models adjusted for sociodemographic factors (age, gender, race/ethnicity, country of origin and education level) and second, in models adjusted for clinical factors (BMI (kg/m^2^), smoking status, diabetes status and NSAID use). Last, adjusted Wald F statistics were estimated to compare follow-up time in months from exam to death between each combination of CMV serostatus and CRP level, adjusting for covariates.

To examine whether more proximal risk factors for CVD, including total cholesterol level and hypertension, lie in the pathway between the combined effect of CMV seropositivity/high CRP level and CVD-related mortality, we compared the HR for CVD-related mortality for those CMV seropositive with high CRP level to individuals that were CMV seronegative with low CRP level, before and after controlling for these factors.

## Results

Weighted estimates of the bivariate relationships between covariates of interest and CMV serostatus are shown in Table 1. The weighted proportion of those seropositive to CMV was 66.7%. Higher mean age, female gender, non-white race/ethnicity, country of origin outside the U.S., lower mean education level, low BMI (<25 kg/m^2^) and high BMI (≥30 kg/m^2^) compared to medium BMI (25≤ BMI <30), reported diabetes and high CRP level (≥0.3 mg/dL) were associated with CMV seropositivity. Although hypothesized as a potential confounder in the association between CMV serostatus and mortality, smoking status was not associated with CMV serostatus in our study. During the mean 13.7 years of follow-up from exam, the population estimate for the proportion dying from all causes was 18.9% and the proportion dying from CVD-related mortality was 8.0%.

**Table 1:** Demographic and Clinical Characteristics (Weighted) by Cytomegalovirus Serostatus Among Subjects Aged 25 and Older in NHANES III.

	CMV Serostatus (N = 14153)		
	Seronegative	Seropositive	
	33.3%	66.7%	
Covariate			p-value
**Age (Years at Examination, range 25**–**90) (Mean ± SE)**	41.3±0.43	51.1±0.51	<.0001*
**Gender**			
Female	44.3%	56.1%	<.0001*
Male	55.7%	43.9%	
**Race/Ethnicity**			
Non-Hispanic White	91.7%	71.0%	<.0001*
Non-Hispanic Black	4.1%	13.1%	
Mexican-American	1.6%	6.2%	
Other	2.6%	9.7%	
**Country of Origin** †			
United States	95.7%	82.1%	<.0001*
Other	4.3%	17.9%	
**Education Level (Years)** ‡ **(Mean ± SE)**	13.5±0.09	11.8±0.09	<.0001*
**Body Mass Index (kg/m^2^)** ±			
≤24.9	45.4%	40.8%	.0317*
25–29.9	32.6%	34.9%	
≥30	22.0%	24.3%	
**Smoking Category**			
Never smoker	44.8%	44.1%	.9221
Former smoker	26.8%	28.4%	
Current smoker	28.4%	27.5%	
**Diabetes** §			
No	96.0%	93.0%	<.0001*
Yes	4.0%	7.0%	
**C-reactive Protein Level**			
Low (<0.3 mg/dL)	76.1%	67.0%	<.0001*
High (≥0.3 mg/dL)	23.9%	33.0%	

*Significant at p<0.05, t-tests for difference in means and Pearson chi-square tests for differences in proportions or test for trend among demographic groupings were calculated.

† N = 14114 due to 39 subjects missing data on country of origin.

‡ N = 14059 due to 94 subjects missing data on education level.

± N = 14118 due to 35 subjects missing data on body mass index (kg/m^2^).

§ N = 14139 due to 14 subjects missing data on diabetes status.

**Figure 1:**
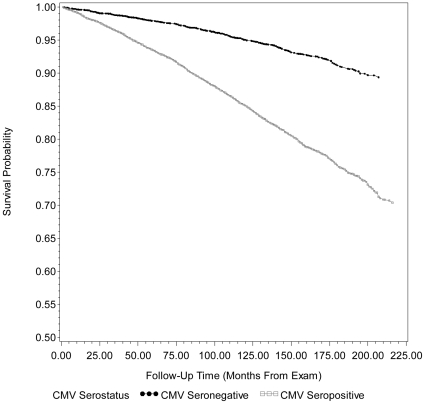
Kaplan-Meier survival curve for all-cause mortality by cytomegalovirus serostatus. Unadjusted Kaplan-Meier survival curves for all-cause mortality by cytomegalovirus (CMV) serostatus for 14153 subjects, ≥25 years of age, in the National Health and Nutrition Examination Survey (NHANES) III from 1988–2006. After adjusting for age, gender, race/ethnicity, country of origin, education level, BMI (kg/m^2^), smoking status and diabetes status, follow-up time from exam to death from all causes was significantly different between CMV seronegative and CMV seropositive subjects (Adjusted Wald F  = 4.66, p-value  = 0.0358). CMV = cytomegalovirus and MEC = mobile examination center.

**Figure 2:**
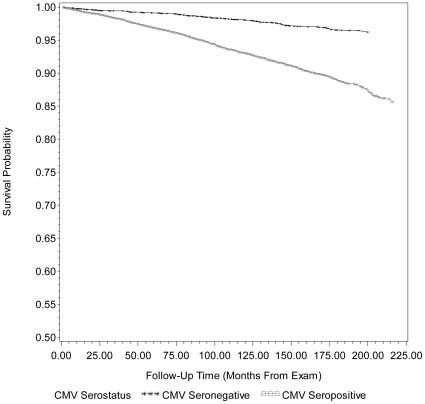
Kaplan Meier survival curve for cardiovascular disease-related mortality by cytomegalovirus serostatus. Unadjusted Kaplan-Meier survival curves for cardiovascular disease (CVD)-related mortality by cytomegalovirus (CMV) serostatus for 14105 subjects, ≥25 years of age, in the National Health and Nutrition Examination Survey (NHANES) III from 1988–2006. After adjusting for age, gender, race/ethnicity, country of origin, education level, BMI (kg/m^2^), smoking status and diabetes status, follow-up time from exam to death from CVD was not significantly different between CMV seronegative and CMV seropositive subjects (Adjusted Wald F  = 2.66, p-value  = 0.1092. CMV = cytomegalovirus and MEC = mobile examination center.

The unadjusted Kaplan-Meier survival curves for all-cause mortality by CMV serostatus are shown in Figures 1 and 2. Overall mean survival duration since time of exam for those CMV seropositive was 13.4 years (160.4±2.40 months) whereas mean survival duration was 14.5 years (173.5±3.15 months) for CMV seronegative subjects. Table S1. shows the HR and 95% CI from Cox proportional hazard models examining the association between CMV and all-cause/CVD-related mortality, mutually adjusted for hypothesized confounders. After adjusting for age, gender, race/ethnicity, country of origin, education level, BMI (kg/m^2^), smoking status and diabetes status, CMV seropositivity remained statistically significantly associated with all-cause mortality (HR 1.19, 95% CI: 1.01, 1.41) and follow-up time from exam to death from all causes was significantly different between CMV seronegative and CMV seropositive subjects (Adjusted Wald F  = 4.66, p-value  = 0.0358) in model 2. The effect of CMV on all-cause mortality was not further attenuated after controlling for CRP level (HR 1.19, 95% CI: 1.01, 1.40 versus HR 1.19, 95% CI: 1.01, 1.41). Although the magnitude of effect for CVD-related mortality was similar to that of all-cause mortality, the association between CMV and CVD-related mortality was not statistically significant in the fully adjusted model and follow-up time from exam to death from CVD-related mortality for those CMV seropositive versus seronegative was not statistically significantly different (Adjusted Wald F  = 2.66, p-value  = 0.1092). Similarly, adjustment for CRP level did not further attenuate the association between CMV and CVD-related mortality (HR 1.19, 95% CI: 0.95, 1.49 versus HR 1.19, 95% CI: 0.96, 1.49).

**Figure 3:**
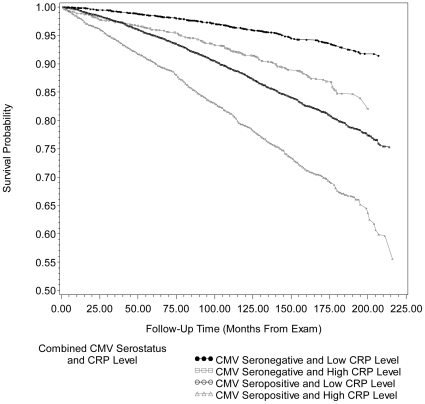
Kaplan-Meier survival curve for all-cause mortality by combined cytomegalovirus serostatus and c-reactive protein level. Unadjusted Kaplan-Meier survival curves for all-cause mortality by combined cytomegalovirus (CMV) serostatus and C-reactive Protein (CRP) level for 14011 subjects, ≥25 years of age, in the National Health and Nutrition Examination Survey (NHANES) III from 1988–2006. After adjusting for age, gender, race/ethnicity, country of origin, education level, BMI (kg/m^2^), smoking status, diabetes status and non-steroidal anti-inflammatory drug use, follow-up time from exam to death from all-causes for CMV seropositive individuals with high CRP level was significantly different from CMV seropositive individuals with low CRP level (Adjusted Wald F  = 36.19, p<0.0001). CMV = cytomegalovirus, CRP = C-reactive Protein and MEC = mobile examination center. High CRP level: ≥0.3 mg/dL.

**Figure 4:**
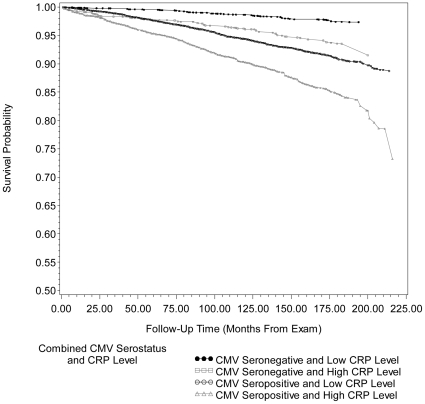
Kaplan-Meier survival curve for cardiovascular disease-related mortality by combined cytomegalovirus serostatus and c-reactive protein level. Unadjusted Kaplan-Meier survival curves for cardiovascular disease (CVD)-related mortality by combined cytomegalovirus (CMV) serostatus and C-reactive Protein (CRP) level for 13963 subjects, ≥25 years of age, in the National Health and Nutrition Examination Survey (NHANES) III from 1988–2006. After adjusting for age, gender, race/ethnicity, country of origin, education level, BMI (kg/m^2^), smoking status, diabetes status and non-steroidal anti-inflammatory drug use, follow-up time from exam to CVD-related death for CMV seropositive individuals with high CRP level was significantly different from CMV seropositive individuals with low CRP level (Adjusted Wald F  = 9.10, p = 0.0040). CMV = cytomegalovirus, CRP = C-reactive Protein and MEC = mobile examination center. High CRP level: ≥0.3 mg/dL.

Figures 3 and 4 show the unadjusted Kaplan-Meier survival curves for all-cause and CVD-related mortality by the four different permutations of CMV serostatus and CRP level. The overall mean survival times from exam to mortality for each combination of CMV serostatus and CRP level were 14.7 years (176.0±3.20 months) for CMV seronegative individuals with low CRP, 13.8 years (165.6±3.49 months) for CMV seronegative individuals with high CRP, 13.8 years (165.5±2.46 months) for CMV seropositive individuals with low CRP and 12.5 years (150.0±3.49 months) for CMV seropositive individuals with high CRP.

Table 2 shows the HR and 95% CI from Cox proportional hazard models examining the association between different permutations of CMV serostatus and CRP level and all-cause or CVD-related mortality, adjusted for confounders. After adjustment for age, gender, race/ethnicity, country of origin, education level, BMI (kg/m^2^), smoking status, diabetes status and NSAID use, the highest HR for all-cause and CVD-related mortality was found among people who were both CMV seropositive and had high CRP levels (HR 1.60, 95% CI: 1.31, 1.94, and HR 1.71, 95% CI: 1.21, 2.42, respectively), compared to CMV seronegative subjects with low CRP. Hence, even after confounder adjustment, subjects that were CMV seropositive and had high CRP had a 30.1% higher risk for all-cause mortality and 29.5% higher risk for CVD-related mortality compared to CMV seropositive subjects who had low CRP levels. The follow-up time from exam to death from all-causes for CMV seropositive individuals with high CRP was significantly different from CMV seropositive individuals with low CRP (Adjusted Wald F  = 36.19, p<0.0001) in fully adjusted models. In addition, the follow-up time from exam to CVD-related death for CMV seropositive individuals with high CRP was significantly different from CMV seropositive individuals with low CRP (Adjusted Wald F  = 9.10, p = 0.0040) in fully adjusted models. After adjustment for total cholesterol level and hypertension, the HR for CVD-related mortality among those CMV seropositive with high CRP level compared to individuals that were CMV seronegative with low CRP, was attenuated by 3.5% (HR 1.65 95% CI: 1.16, 2.35, compared to HR 1.71, 95% CI: 1.21, 2.42).

**Table 2:** The Combined effect of Cytomegalovirus (CMV) Serostatus and C-reactive Protein (CRP) Level on All-Cause/Cardiovascular Disease (CVD) -related Mortality in Subjects 25 Years of Age and Older in NHANES III.

	Hazard Ratio (95% Confidence Interval)			
	All-Cause Mortality		CVD-related Mortality	
	Model 1†	Model 2‡	Model 1†	Model 2‡
Combined Factors				
CMV Seronegative and Low CRP Level	1.0	1.0	1.0	1.0
CMV Seronegative and High CRP Level	1.56 (1.23, 1.97)*	1.46 (1.16, 1.83)*	1.81 (1.17, 2.78)*	1.67 (1.08, 2.57)*
CMV Seropositive and Low CRP Level	1.25 (1.04, 1.49)*	1.23 (1.03, 1.47)*	1.33 (0.97, 1.81)	1.32 (0.97, 1.78)
	1.73 (1.41, 2.11)*	1.60 (1.31, 1.94)*	1.85 (1.31, 2.60)*	1.71 (1.21, 2.42)*

*Significant at p<0.05.

† Model 1 for all-cause mortality (N = 14029) and for CVD-related mortality (N = 136981) adjusted for age, gender, race/ethnicity, country of origin and education level and reduced by 124 subjects due to missing data on country of origin and/or education level.

‡ Model 2 for all-cause mortality (N = 13611) and for CVD-related mortality (N = 13569) adjusted for age, gender, race/ethnicity, country of origin, education level, body mass index (kg/m^2^), smoking status, diabetes status and non-steroidal anti-inflammatory drug use. Model 2 for all-cause mortality reduced by an additional 418 subjects and model 2 for CVD-related mortality reduced by an additional 322 subjects due to missing data on body mass index, smoking status, diabetes status and/or non-steroidal anti-inflammatory drugs.

## Discussion

This study examined whether seropositivity for CMV, an indicator of prior infection with this persistent herpesvirus, predicts all-cause as well as CVD-related mortality and whether CRP mediates or modifies this relationship in a nationally representative U.S. population of individuals 25 years of age and older. The results of our study suggest that CMV seropositivity is independently associated with all-cause mortality, after controlling for age, gender, race/ethnicity, country of origin, education level, BMI (kg/m^2^), smoking status and diabetes status. Furthermore, adjustment for CRP level did not attenuate this relationship.

After confounder adjustment, CMV serostatus was no longer significantly associated with CVD mortality. It is possible that the strong relationship between CMV and all-cause mortality observed in this study indicates that CMV seropositive subjects are dying of competing causes before they have the chance to develop CVD, attenuating the relationship between CMV and CVD-related mortality. High levels of CRP, however, augmented the effect of CMV seropositivity on both all-cause and CVD-related mortality. Among CMV seropositive subjects, those with high CRP levels showed approximately a 30% higher risk for all-cause mortality and for CVD-related mortality, compared to those with low CRP levels. Adjusting for CVD risk factors such as high total cholesterol and hypertension resulted in a moderate attenuation (decrease of 3.5%) of the association between combined CMV seropositivity and high CRP level and CVD-related mortality, supporting the hypothesis that CMV seropositivity along with subclinical inflammation impacts risk for mortality in part through their combined contribution to other more proximal CVD risk factors. Nonetheless, these markers of CVD did not completely attenuate the association, indicating that other factors may also be on the pathway or that there is a direct relationship between CMV infection, CRP levels and CVD-related mortality. Thus, our research suggests that efforts identifying the mechanisms of these additive effects and targeted CVD intervention studies among subpopulations with these biomarker risk profiles are warranted.

It has been suggested that individuals who are CMV seropositive and also have a subclinical inflammatory profile are more susceptible to the atherogenic effects of CMV infection, whereas those without a subclinical response are not as susceptible [43], [51]. Studies support a correlation between increased CRP and increased CMV antibody titers- a marker of reactivation - but the directionality among these two biological markers is unclear [49], [50], [71]. If elevated CRP is a marker of “reactivating” CMV infection, whereas low CRP indicates resolved or “latent” CMV infection, we would expect CRP levels to be increased during times of CMV reactivation and for CMV infection to be most detrimental under these circumstances. Since CMV has been found to directly invade cardiovascular tissues [21]–[23], periods of CMV “reactivation” may accelerate the atherogenic process by increasing the detrimental effects of tissue invasion such as smooth muscle cell proliferation and migration [16], [17], [25], [26]. On the other hand, if, as hypothesized, molecular mimicry plays a role in the relationship between CMV and CVD-mortality, the immune system may become hyper-stimulated during periods of CMV “reactivation” leading to an exacerbated attack against host tissues presenting cross-reacting human peptides [27]–[30]. For these reasons, the greatest physiological harm caused by CMV is likely to occur during “reactivation” events, possibly at a subclinical level. If subclinical “reactive” CMV infection is most important in the etiology of mortality, then situations that cause CMV reactivation over the lifecourse, such as stress, inflammation caused by co-infections, immunosuppression and aging may play important key roles in determining the extent to which CMV infection is detrimental to health.

Although CMV IgG and IgM antibody titer levels are available in NHANES III, values for subjects over age 49 were top-coded, making them unusable as a predictor of mortality. It was also not possible to correlate antibody levels as an indicator of “reactivating” versus “latent” CMV infection in our study population. In addition, approximately 64% of our study population had CRP levels under the limit of detection (<0.3 mg/dl), because more sensitive assays that are currently available for measurement of high-sensitivity CRP below this limit of detection were not used in NHANES III, making it difficult to examine CRP levels continuously. Hence, one limitation of our study is that were unable to examine the relationship between CMV antibody titer and CRP level more closely, which might have allowed us to identify a more precise interaction between CMV infection and CRP levels and their effect on all-cause and CVD-related mortality. Another limitation associated with using CRP as a predictor of mortality is that very high values of CRP may represent acute infection or injury not necessarily associated with cardiovascular disease. In addition, the causal relations and therapeutic implications pertaining to CRP and CVD are currently unclear [72]–[75]. However, other inflammatory markers that have been shown to play a significant role in the pathway between CMV and mortality, such as IL-6, are not available in NHANES III. Thus, it was not possible to determine whether other inflammatory markers in addition to CRP mediate or modify the relationship between CMV and mortality. Nonetheless, our exposure of interest, CMV serostatus, was laboratory-confirmed and CRP was measured by standard methodologies [1], [53], [55].

Another limitation to our study is that we utilized the updated NHANES III Linked Mortality Public-use File which was subject to data perturbation in which synthetic data was substituted for the actual date of death and underlying cause of death for selected descendent records in order to reduce risk of respondent re-identification [76]. Despite the data perturbation of date of death for selected decedents in the public-use file, a comparison study between the public-use and restricted-use file (which does not contain perturbed data) conducted by the NCHS, found that analysis of all-cause and cause-specific mortality (heart disease, ischemic heart disease and cerebrovascular disease, in particular) by sociodemographic factors including as age, sex, race/ethnicity and education level to be comparable across files and concluded that the discrepancies between the public-use and restricted-use file were minor and that analysts should use the public-use file with confidence [76]. Importantly, mortality status was rigorously verified by two sources (National Death Index (NDI) and/or death certificate review) [53]. Furthermore, NDI is a validated method for matching deaths in the U.S. to population-based datasets and for obtaining cause of death [77].

Several studies have examined the relationship between CMV and mortality [14], [49]–[52]. However, these earlier studies have only examined the relationship among specific gender and racial/ethnic subgroups, often among subjects with existing CVD and primarily compared mortality by CMV antibody titer level (not CMV serostatus). To the best of our knowledge, our study is the first to utilize a large, nationally representative U.S. population aged 25 and older with up to 18.1 years of follow-up (mean of 13.7 years) to examine the relationship between CMV serostatus, CRP level and all-cause/CVD-related mortality. Therefore, the population studied here is much broader in age and on average younger than the study populations utilized in several earlier studies examining the impact of CMV infection on mortality. A moderate increase in risk among a large population with a wide age range in the US has important implications for potentially shifting the population burden of diseases [78].

If CMV infection plays a strong role in the etiology of all-cause and CVD-related mortality in conjunction with high CRP level, the elimination of CMV infection via the development and administration of treatments or vaccines [79] and/or targeting the interaction of infection and CRP levels may reduce mortality rates in the United States. Colugnati et al. predicted that a vaccination against CMV would not need to have high efficacy nor wide-spread coverage to make a substantial impact on CMV transmission and elimination of CMV from the population has the potential to greatly reduce the incidence of disease attributable to CMV infection [6], [80]. Therefore, elimination of CMV infection is a potentially feasible and important avenue of study for preventing mortality from all-causes and CVD in the United States.

## Supporting Information

Table S1 **The Relationship between Cytomegalovirus Serostatus, C-Reactive Protein Level and All-Cause/Cardiovascular Disease-Related Mortality in Subjects 25 Years of Age and Older in NHANES III.**
(DOC)
