# Supplementary material for: Seropositivity to Cytomegalovirus, Inflammation, All-Cause and Cardiovascular Disease-Related Mortality in the United States
Source: PLoS One. 2011 Feb 17;6(2):e16103. doi: 10.1371/journal.pone.0016103 (PMC3040745; doi:10.1371/journal.pone.0016103)
Supplement: Table S1 — The Relationship between Cytomegalovirus Serostatus, C-Reactive Protein Level and All-Cause/Cardiovascular Disease-Related Mortality in Subjects 25 Years of Age and Older in NHANES III. (DOC) [file pone.0016103.s001.doc]

Table S1. The Relationship Between Cytomegalovirus Serostatus, C-reactive Protein Level and All-Cause/Cardiovascular Disease-related Mortality in Subjects 25 Years of Age and Older in NHANES III.

|  | **Hazard Ratio ( 95% Confidence Interval)** | | | | | |
| --- | --- | --- | --- | --- | --- | --- |
|  | **All-Cause Mortality** | | | **Cardiovascular Disease-Related Mortality** | | |
|  | **Model 1^†^** | **Model 2^‡^** | **Model 3^¶^** | **Model 1^†^** | **Model 2^‡^** | **Model 3^¶^** |
| **Cytomegalovirus** |  |  |  |  |  |  |
| Seronegative | 1.0 | 1.0 | 1.0 | 1.0 | 1.0 | 1.0 |
| Seropositive | 1.20 (1.03, 1.41)* | 1.19 (1.01, 1.41)* | 1.19 (1.01, 1.40)* | 1.20 (0.96, 1.50) | 1.19 (0.96, 1.49) | 1.19 (0.95, 1.49) |
| **Age (years since exam, range 25-90)** | 1.09 (1.09, 1.10)* | 1.10 (1.09, 1.11)* | 1.10 (1.09, 1.10)* | 1.11 (1.11, 1.12)* | 1.12 (1.11, 1.13)* | 1.12 (1.11, 1.13)* |
| **Gender** |  |  |  |  |  |  |
| Female | 1.0 | 1.0 | 1.0 | 1.0 | 1.0 | 1.0 |
| Male | 1.49 (1.35, 1.64)* | 1.40 (1.27, 1.54)* | 1.42 (1.29, 1.57)* | 1.58 (1.39, 1.80)* | 1.54 (1.34, 1.77)* | 1.57 (1.36, 1.80)* |
| **Race/Ethnicity** |  |  |  |  |  |  |
| Non-Hispanic White | 1.0 | 1.0 | 1.0 | 1.0 | 1.0 | 1.0 |
| Non-Hispanic Black | 1.27 (1.12, 1.45)* | 1.20 (1.05, 1.37)* | 1.17 (1.03, 1.33)* | 1.20 (1.00, 1.44) | 1.11 (0.92, 1.35) | 1.09 (0.90, 1.30) |
| Mexican-American | 0.88 (0.74, 1.05) | 0.88 (0.74, 1.05) | 0.87 (0.73, 1.03) | 0.89 (0.67, 1.17) | 0.86 (0.66, 1.13) | 0.85 (0.65, 1.12) |
| Other | 0.83 (0.64, 1.07) | 0.76 (0.59, 0.99)* | 0.78 (0.60, 1.01) | 0.88 (0.53, 1.47) | 0.78 (0.45, 1.35) | 0.80 (0.47, 1.37) |
| **Country of Origin** |  |  |  |  |  |  |
| United States | 1.0 | 1.0 | 1.0 | 1.0 | 1.0 | 1.0 |
| Other | 0.69 (0.57, 0.84)* | 0.77 (0.64, 0.93)* | 0.78 (0.65, 0.93)* | 0.64 (0.48, 0.85)* | 0.70 (0.53, 0.91)* | 0.70 (0.54, 0.91)* |
| **Education Level (years)** | 0.95 (0.94, 0.97)* | 0.96 (0.95, 0.98)* | 0.97 (0.95, 0.98)* | 0.96 (0.95, 0.97)* | 0.97 (0.96, 0.98)* | 0.97 (0.96, 0.99)* |
| **Body Mass Index (kg/m^2^)** |  |  |  |  |  |  |
| < 25 | ---- | 1.18 (1.04, 1.33)* | 1.22 (1.07, 1.38)* | ---- | 1.14 (0.97, 1.34) | 1.18 (1.00, 1.39) |
| 25 ≤ BMI < 30 | ---- | 1.0 | 1.0 | ---- | 1.0 | 1.0 |
| ≥ 30 | ---- | 1.21 (1.05, 1.40)* | 1.16 (1.00, 1.33) | ---- | 1.34 (1.08, 1.66)* | 1.28 (1.03, 1.58)* |
| **Smoking Status** |  |  |  |  |  |  |
| **Never** | ---- | 1.0 | 1.0 |  | 1.0 | 1.0 |
| **Former** | ---- | 1.28 (1.14, 1.44)* | 1.27 (1.13, 1.42)* |  | 1.16 (1.00, 1.35) | 1.15 (1.00, 1.33) |
| **Current** | ---- | 2.27 (2.00, 2.58)* | 2.20 (1.93, 2.50)* |  | 1.96 (1.62, 2.37)* | 1.90 (1.58, 2.29)* |
| **Diabetes** |  |  |  |  |  |  |
| No | ---- | 1.0 | 1.0 | ---- | 1.0 | 1.0 |
| Yes | ---- | 1.90 (1.65, 2.19)* | 1.87 (1.63, 2.15)* | ---- | 1.99 (1.62, 2.46)* | 1.96 (1.60, 2.42)* |
| **C-reactive Protein Level** |  |  |  |  |  |  |
| Low (< 0.3 mg/dL) | ---- | ---- | 1.0 | ---- | ---- | 1.0 |
| High (≥ 0.3 mg/dL) | ---- | ---- | 1.34 (1.23, 1.45)* | ---- | ---- | 1.36 (1.17, 1.59)* |

*Significant at p<0.05

^†^Model 1 for all-cause mortality (N=14029) and for cardiovascular disease-related mortality (N=13981) adjusted for age, gender, race/ethnicity, country of origin and education level and reduced by 124 subjects due to missing data on country of origin and/or education level.

**^‡^** Model 2 for all-cause mortality (N=13981) and for cardiovascular disease-related mortality (N=13934) adjusted for age, gender, race/ethnicity, country of origin, education level, body mass index (kg/m^2^), smoking status and/or diabetes status. Model 2 for all-cause mortality reduced by and additional 48 subjects and for cardiovascular disease-related mortality reduced by an additional 47 subjects due to missing data on body mass index, smoking status and/or diabetes status.

^¶^ Model 3 for all-cause mortality (N=13981) and for cardiovascular disease-related mortality (N=13934) adjusted for age, gender, race/ethnicity, country of origin, education level, body mass index (kg/m^2^), smoking status, diabetes status and C-reactive protein level.
